# Supplementary material for: Norsesquiterpenes from the Latex of Euphorbia dentata and Their Chemical Defense Mechanisms against Helicoverpa armigera
Source: Molecules. 2023 Nov 21;28(23):7681. doi: 10.3390/molecules28237681 (PMC10707868; doi:10.3390/molecules28237681)
Supplement: Supplementary file 1 [file molecules-28-07681-s001.zip › molecules-2690577-supplementary.pdf]

Supporting Information

Figure S1. The HR-ESIMS spectrum of compound **1**

Figure S2. The ^{1}H-NMR spectrum of compound **1**

Figure S3. The ^{13}C-NMR spectrum of compound **1**

Figure S4. The HSQC spectrum of compound **1**

Figure S5. The HMBC spectrum of compound **1**

Figure S6. The HR-ESIMS spectrum of compound **2**

Figure S7. The ^{1}H-NMR spectrum of compound **2**

Figure S8. The ^{13}C-NMR spectrum of compound **2**

Figure S9. The HSQC spectrum of compound **2**

Figure S10. The HMBC spectrum of compound **2**

Figure S11. The HR-ESIMS spectrum of compound **3**

Figure S12. The ^{1}H-NMR spectrum of compound **3**

Figure S13. The ^{13}C-NMR spectrum of compound **3**

Figure S14. The HSQC spectrum of compound **3**

Figure S15. The HMBC spectrum of compound **3**

Figure S16. The ^{1}H-NMR spectrum of compound **4**

Figure S17. The ^{13}C-NMR spectrum of compound **4**

Figure S18. The ^{1}H-NMR spectrum of compound **5**

Figure S19. The ^{13}C-NMR spectrum of compound **5**

Figure S20. The ^{1}H-NMR spectrum of compound **6**

Figure S21. The ^{13}C-NMR spectrum of compound **6**

Figure S22. The ^{1}H-NMR spectrum of compound **7**

Figure S23. The ^{13}C-NMR spectrum of compound **7**

Figure S24. The ^{1}H-NMR spectrum of compound **8**

Figure S25. The ^{13}C-NMR spectrum of compound **8**

Figure S26. The ^{1}H-NMR spectrum of compound **9**

Figure S27. The ^{13}C-NMR spectrum of compound **9**

Figure S28. The ^{1}H-NMR spectrum of compound **10**

Figure S29. The ^{13}C-NMR spectrum of compound **10**

Figure S30. The superimposed 3D structures of GST target (red) and template (PDB code: 3VK9) (blue)

Figure S31. Antifeedant effects of the latex of *E. dentata* on *H. armigera*

Figure S32. Antifeedant effects of the methanol extract of latex on *H. armigera*

Figure S33. The flowchart of extraction and isolation of the latex of *E. dentata*

Table S1. The optimal template for 3D structure modelling of GST

Table S2. Moldock scores of the compounds (**1**−**3**) with GST of *H. armigera*

Figure S1.

Mass Spectrum Molecular Formula Report

Analysis Info

Analysis Name	D:\Data\20230610\Comp.1.d	Acquisition Date	06/10/2023 3:34:11 PM
Method	danticeshi.m	Instrument / Ser#	Bruker Customer
Sample Name	Comp.1	Operator	micrOTOF-Q 125
Comment			

Acquisition Parameter

Source Type	ESI	Ion Polarity	Positive	Set Nebulizer	1.2 Bar
Focus	Active	Set Capillary	4500 V	Set Dry Heater	180 °C
Scan Begin	50 m/z	Set End Plate Offset	-500 V	Set Dry Gas	8.0 l/min
Scan End	3000 m/z	Set Collision Cell RF	400.0 Vpp	Set Divert Valve	Source

Generate Molecular Formula Parameter

Formula, min.		
Formula, max.		
Measured m/z	Tolerance	Charge
Check Valence	Minimum	Maximum
Nirogen Rule	Electron Configuration	
Filter H/C Ratio	Minimum	Maximum
Estimate Carbon		

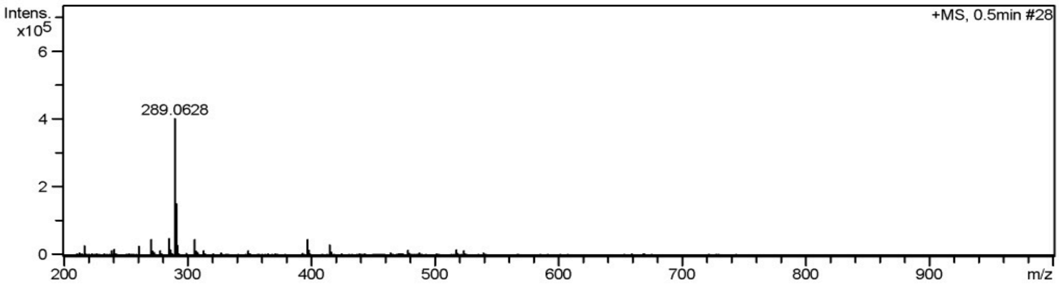

Figure S2.

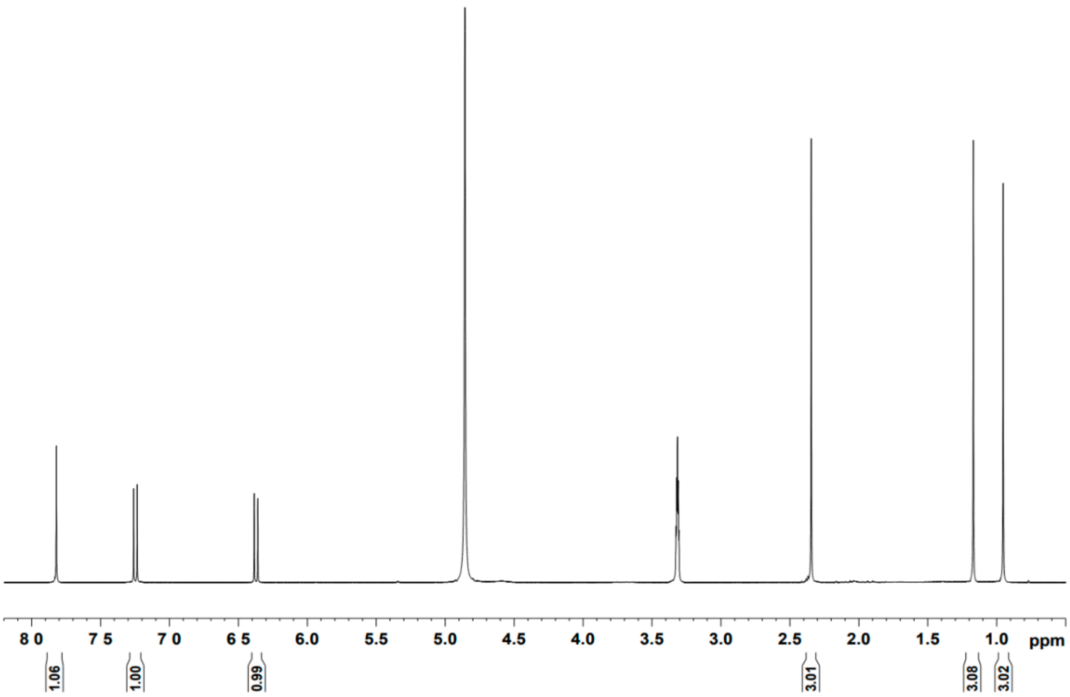

Figure S3.

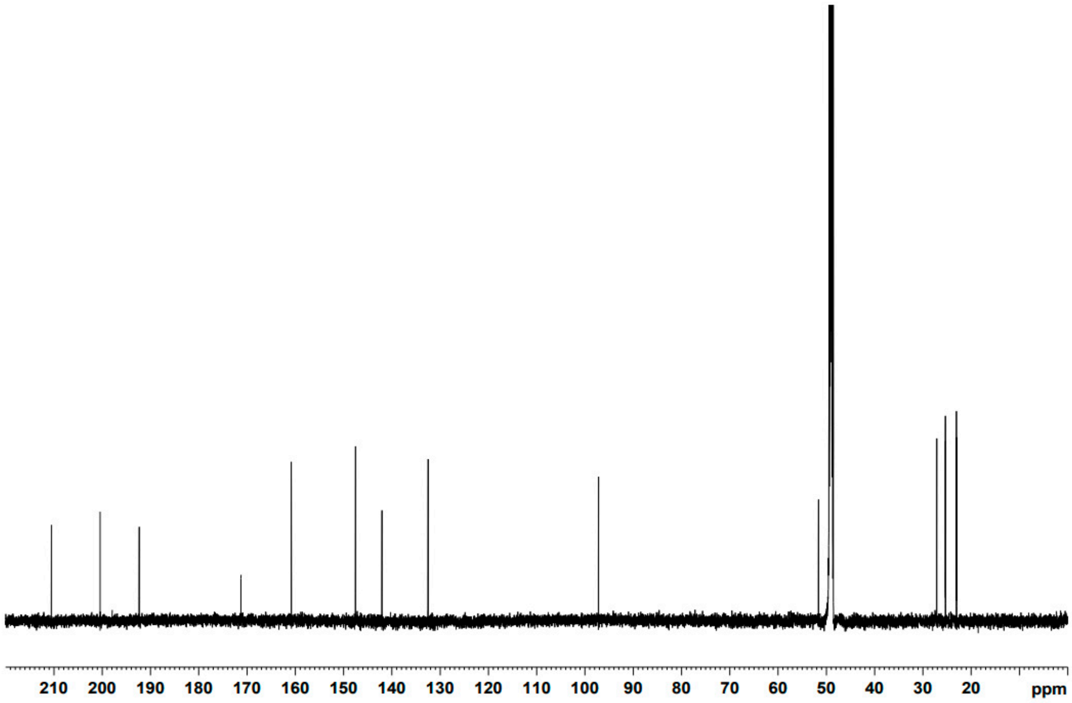

Figure S4.

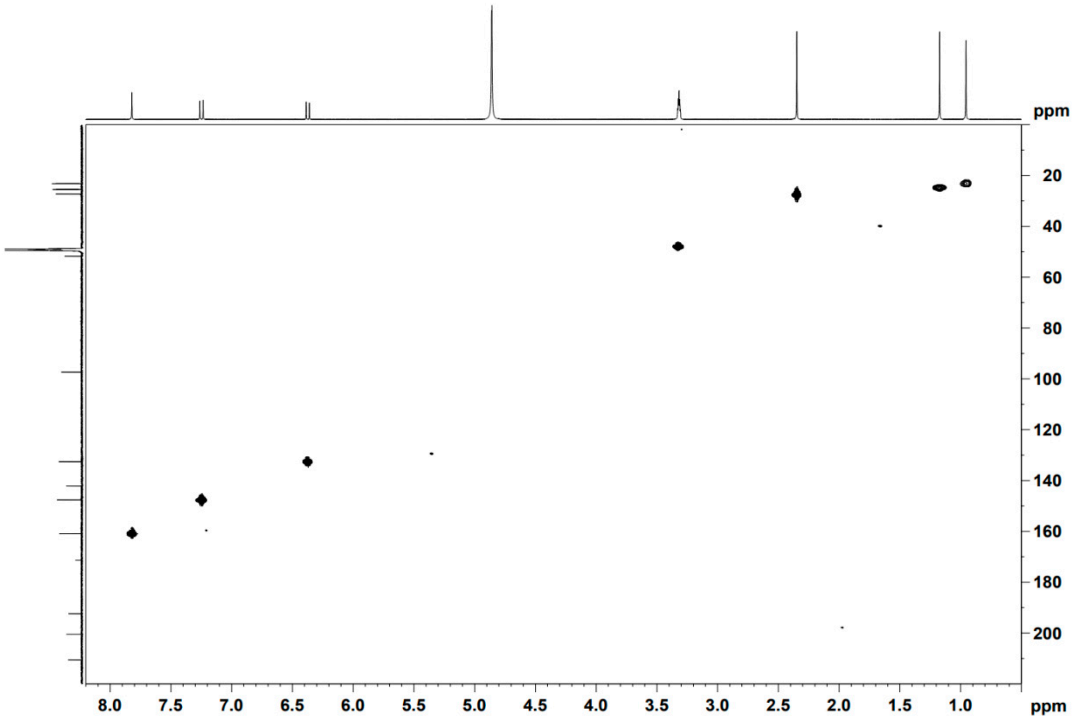

Figure S5.

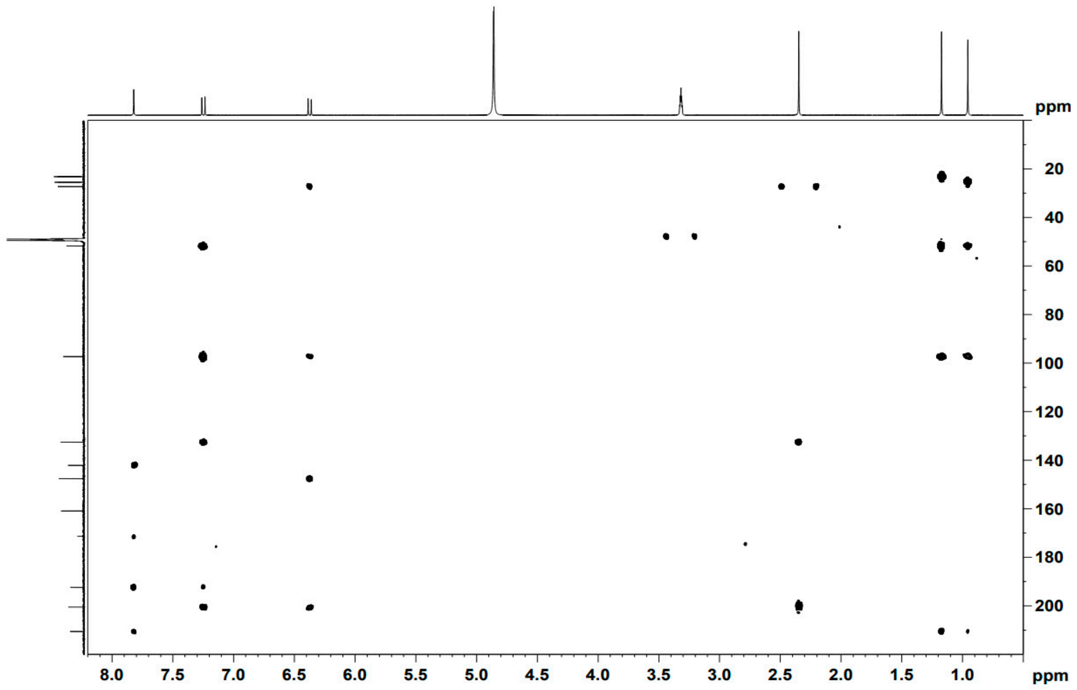

Figure S6.

Mass Spectrum Molecular Formula Report

Analysis Info

Analysis Name	D:\Data\20230610\Comp.2.d	Acquisition Date	06/10/2023 1:26:49 PM
Method	danticeshi.m	Instrument / Ser#	Bruker Customer
Sample Name	Comp.2	Operator	micrOTOF-Q 125
Comment			

Acquisition Parameter

Source Type	ESI	Ion Polarity	Positive	Set Nebulizer	1.2 Bar
Focus	Active	Set Capillary	4500 V	Set Dry Heater	180 °C
Scan Begin	50 m/z	Set End Plate Offset	-500 V	Set Dry Gas	8.0 l/min
Scan End	3000 m/z	Set Collision Cell RF	300.0 Vpp	Set Divert Valve	Source

Generate Molecular Formula Parameter

Formula, min.		
Formula, max.		
Measured m/z	Tolerance	Charge
Check Valence	Minimum	Maximum
Nirogen Rule	Electron Configuration	
Filter H/C Ratio	Minimum	Maximum
Estimate Carbon		

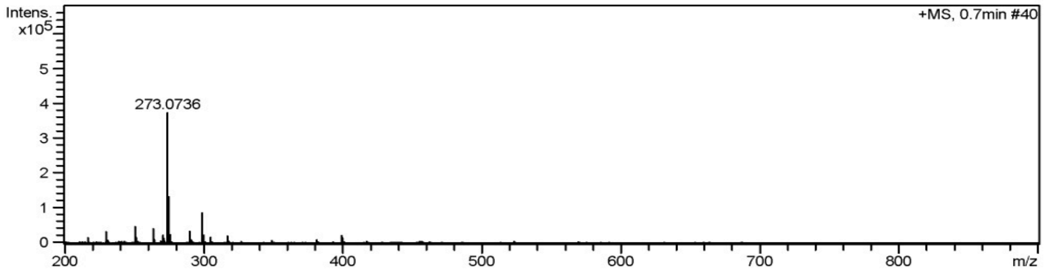

Figure S7.

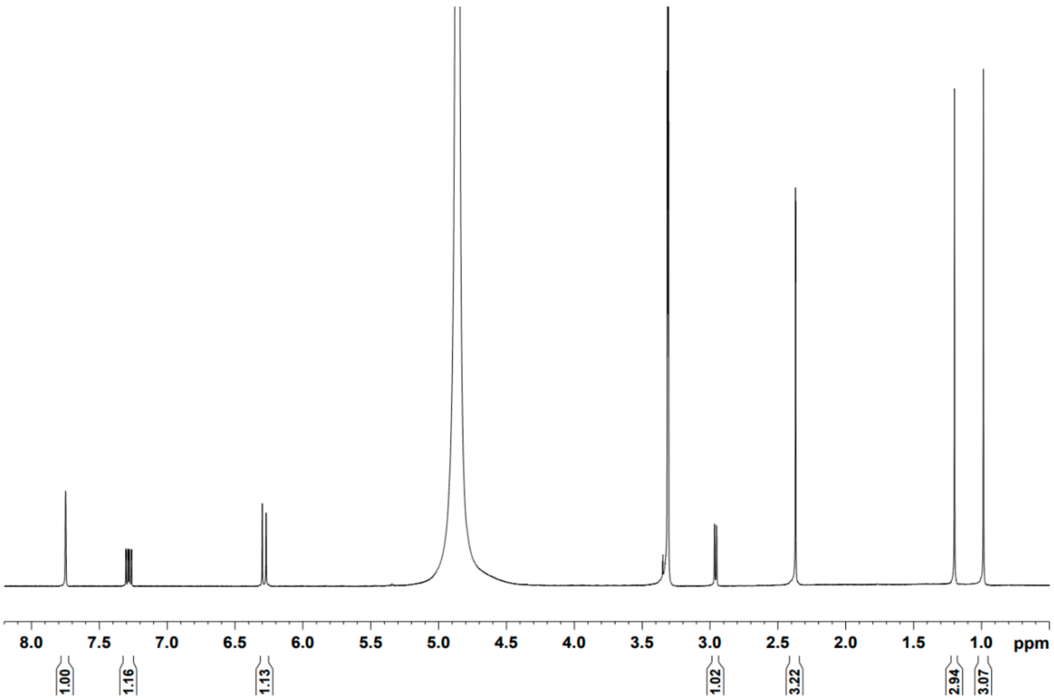

Figure S8.

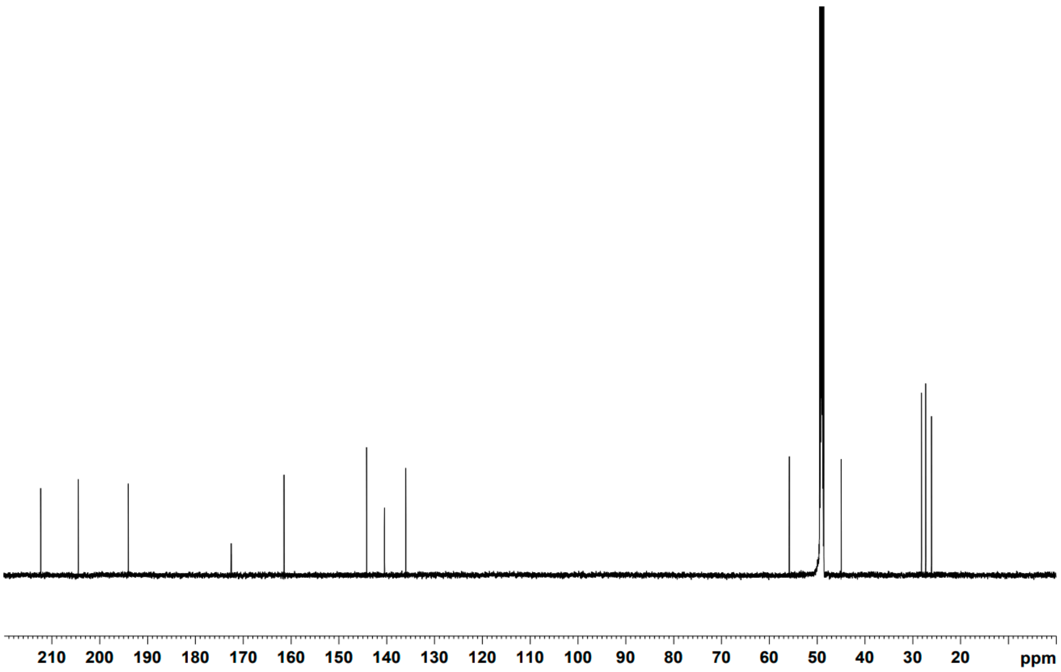

Figure S9.

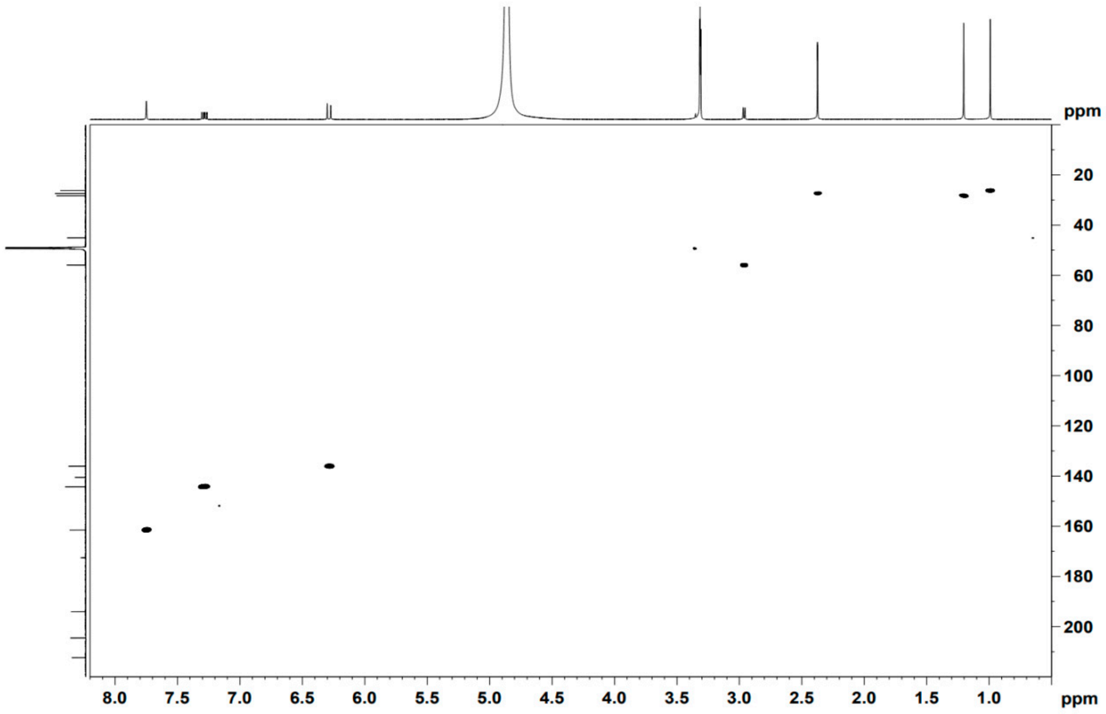

Figure S10.

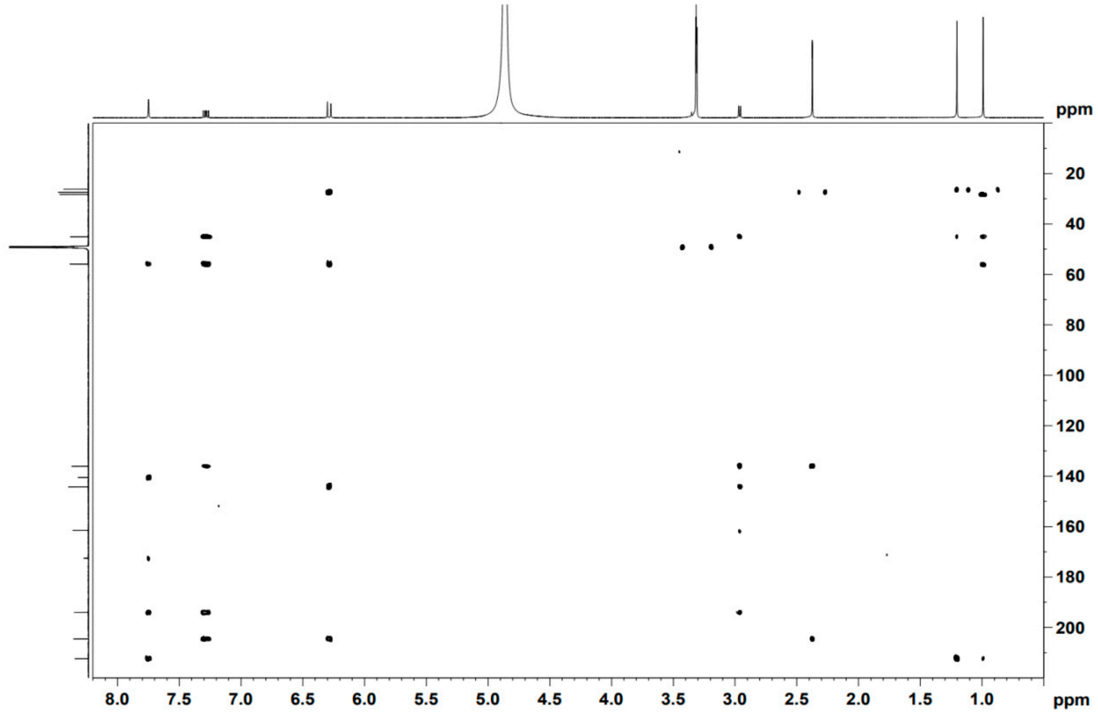

Figure S11.

Mass Spectrum Molecular Formula Report

Analysis Info

Analysis Name	D:\Data\20230610\Comp.3.d	Acquisition Date	06/10/2023 1:26:32 PM
Method	danticeshi.m	Instrument / Ser#	Bruker Customer
Sample Name	Comp.3	Operator	micrOTOF-Q 125
Comment			

Acquisition Parameter

Source Type	ESI	Ion Polarity	Positive	Set Nebulizer	1.2 Bar
Focus	Active	Set Capillary	4500 V	Set Dry Heater	180 °C
Scan Begin	50 m/z	Set End Plate Offset	-500 V	Set Dry Gas	8.0 l/min
Scan End	3000 m/z	Set Collision Cell RF	300.0 Vpp	Set Divert Valve	Source

Generate Molecular Formula Parameter

Formula, min.		
Formula, max.		
Measured m/z	Tolerance	Charge
Check Valence	Minimum	Maximum
Nirogen Rule	Electron Configuration	
Filter H/C Ratio	Minimum	Maximum
Estimate Carbon		

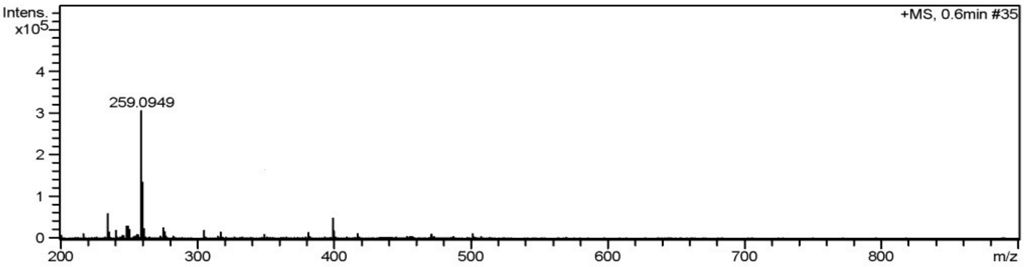

Figure S12.

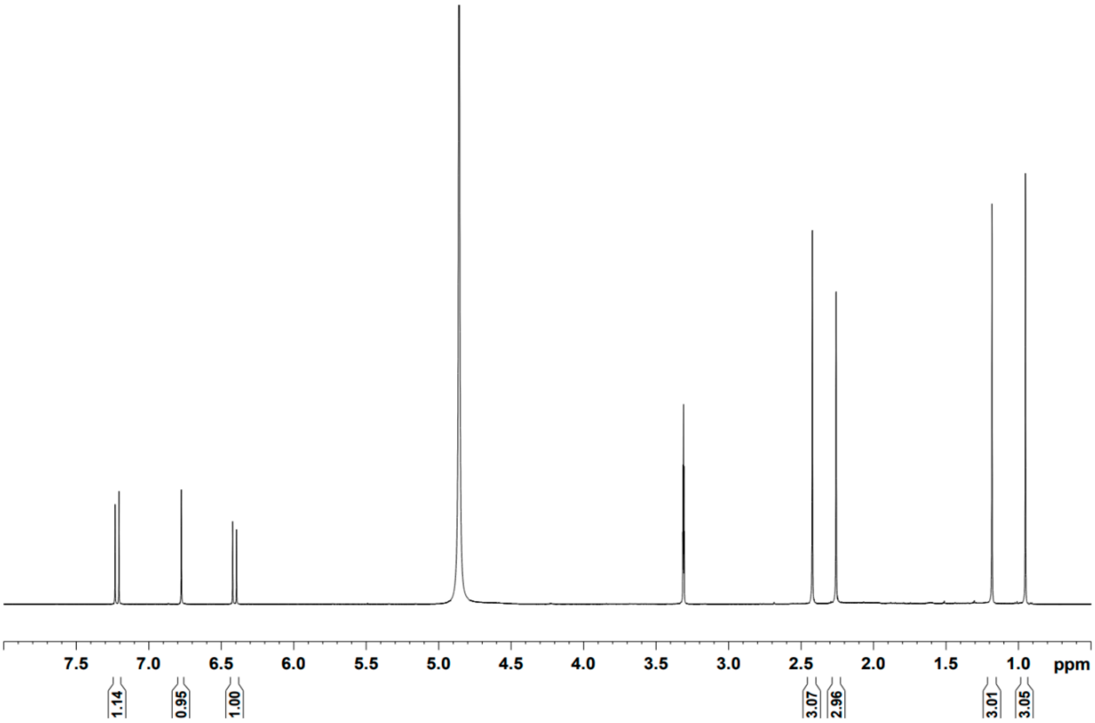

Figure S13.

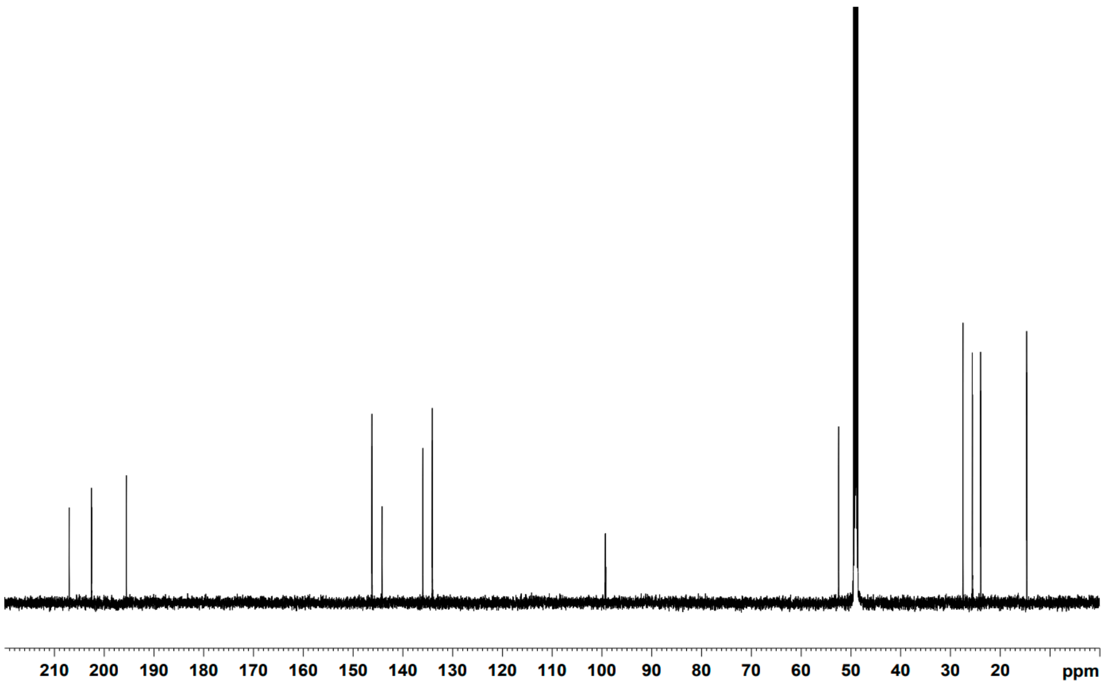

Figure S14.

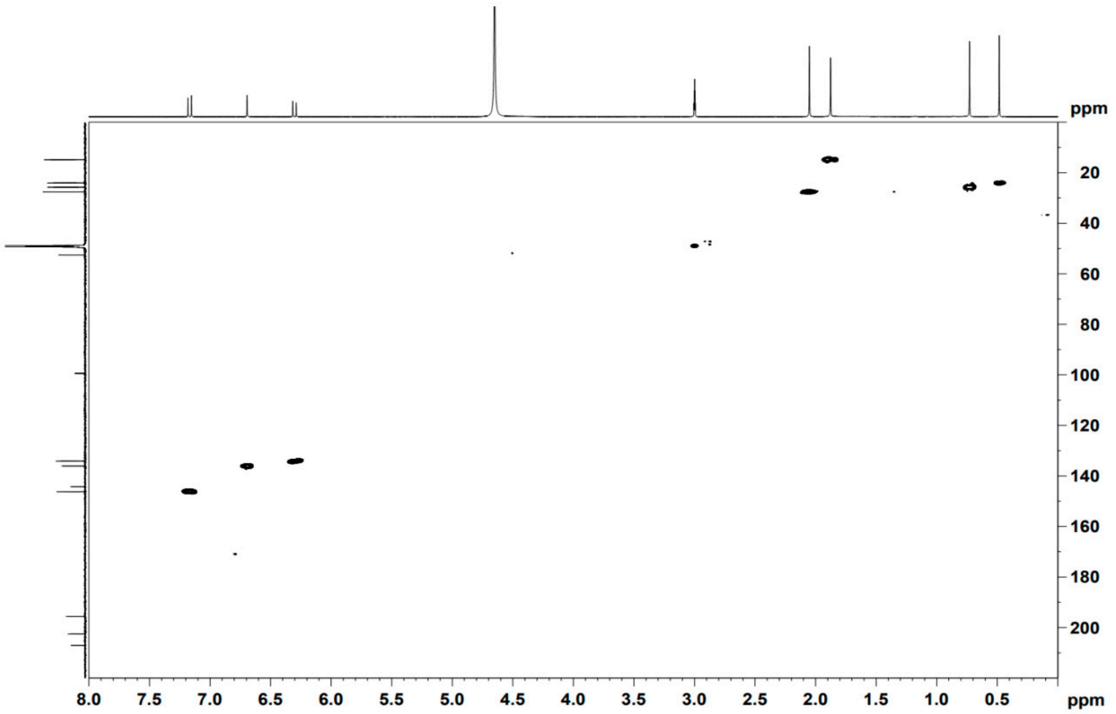

Figure S15.

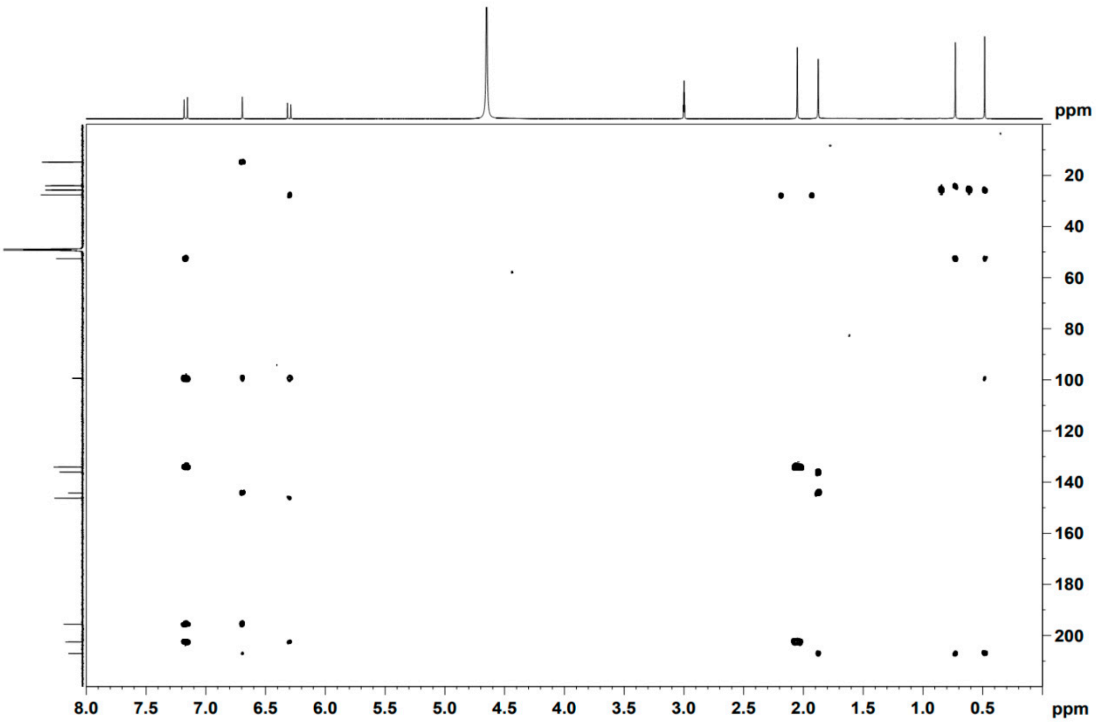

Figure S16.

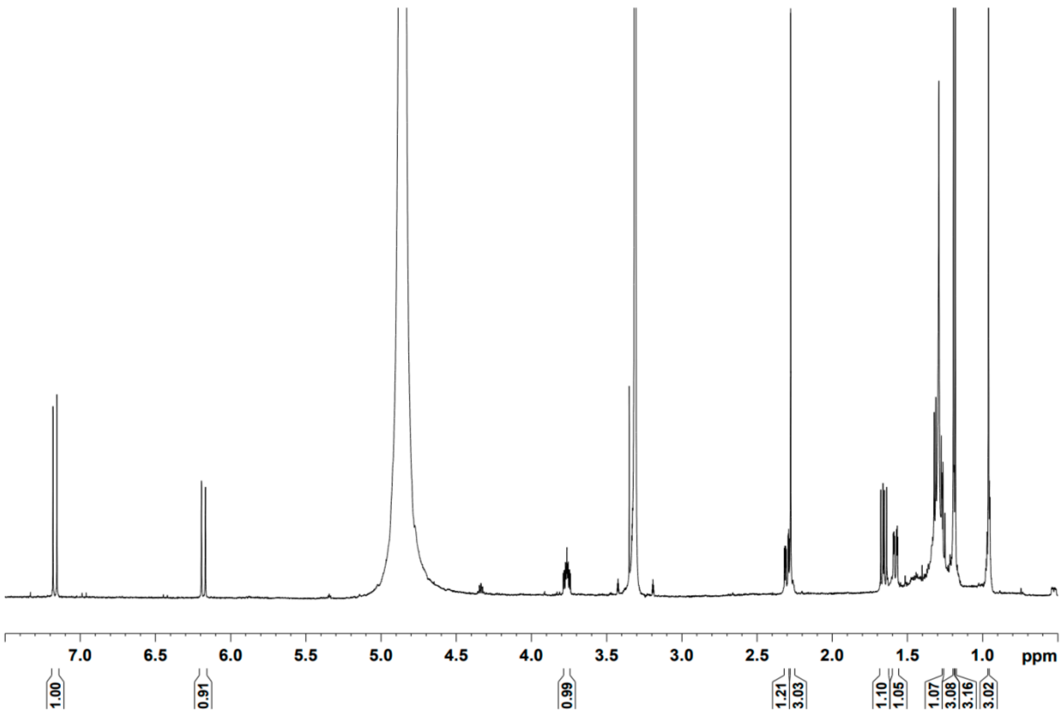

Figure S17.

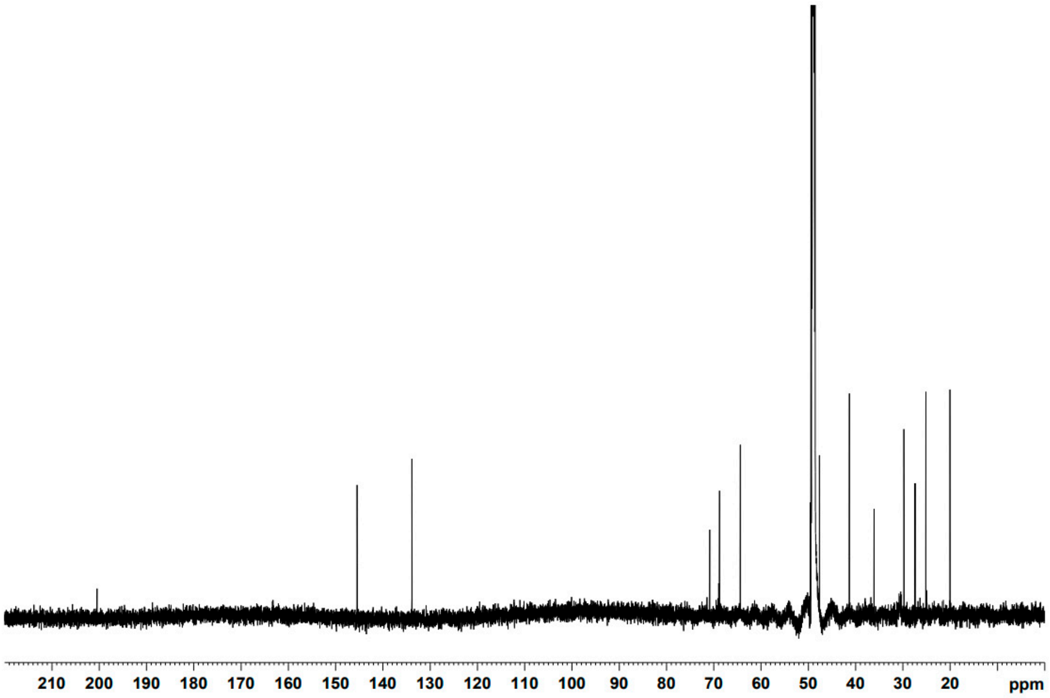

Figure S18.

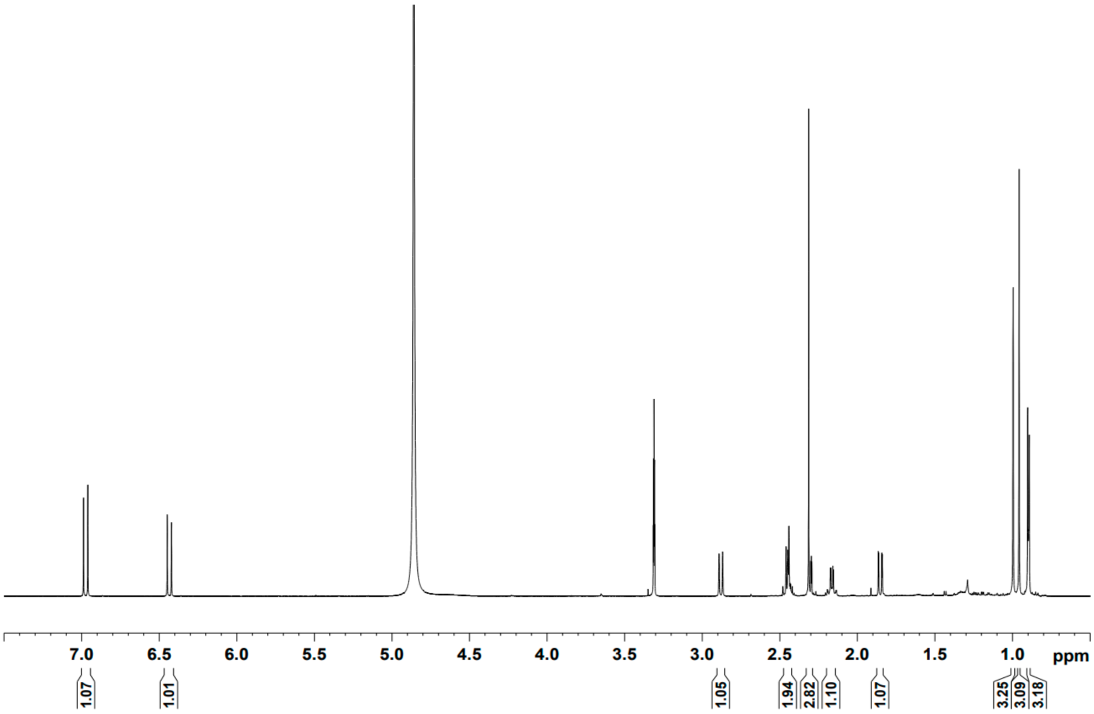

Figure S19.

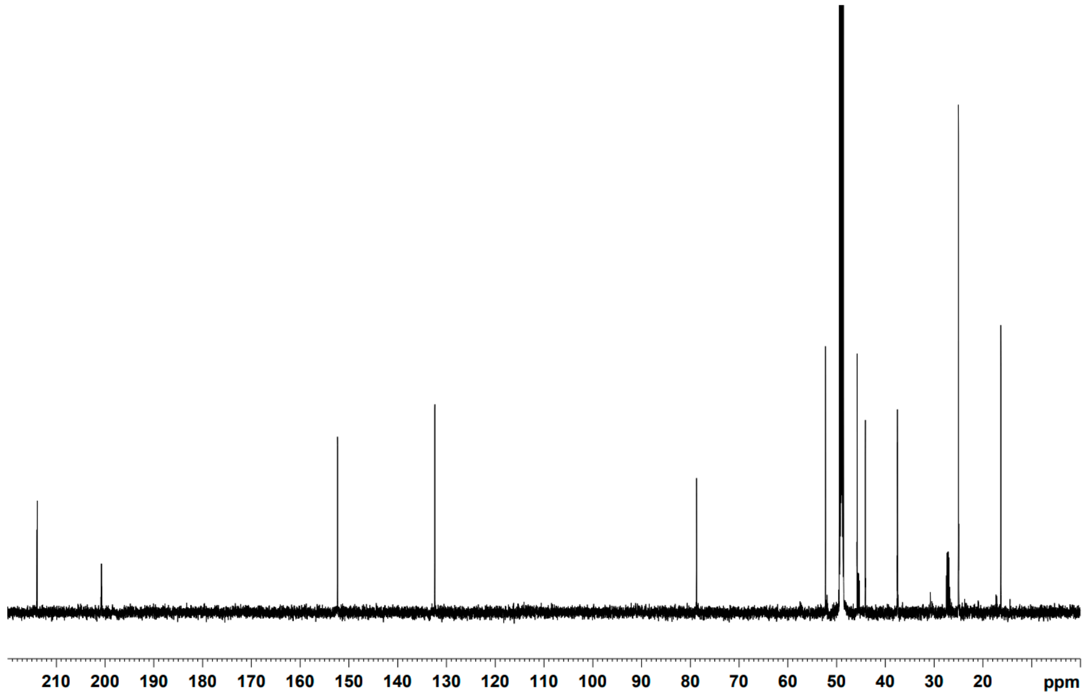

Figure S20.

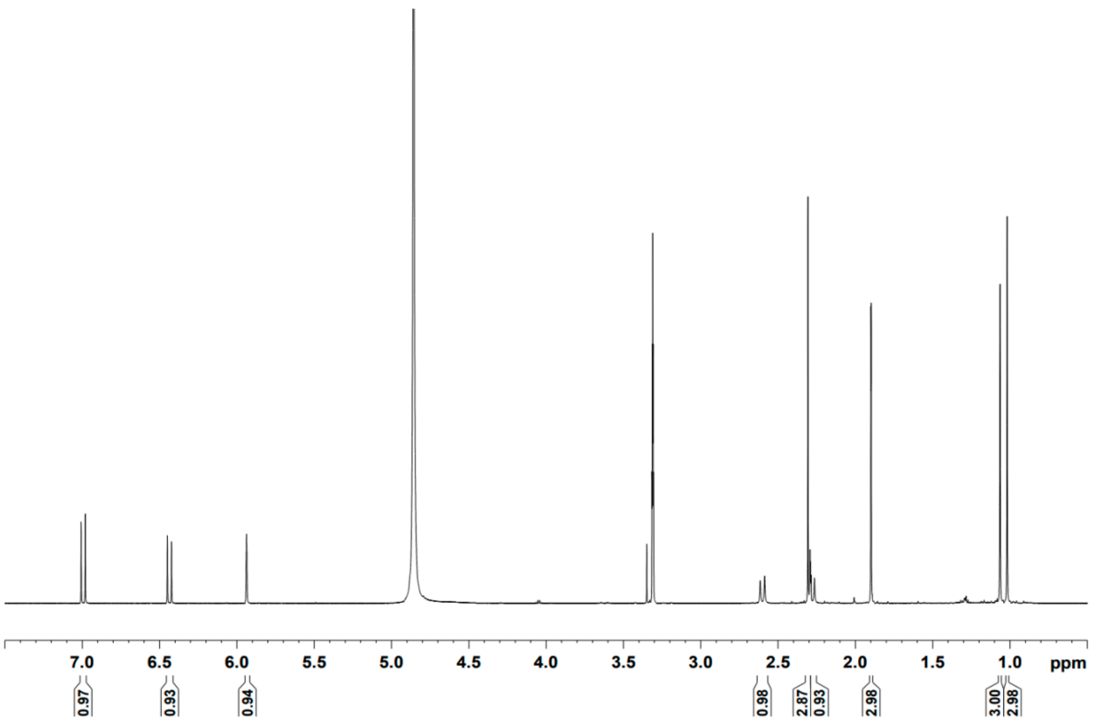

Figure S21.

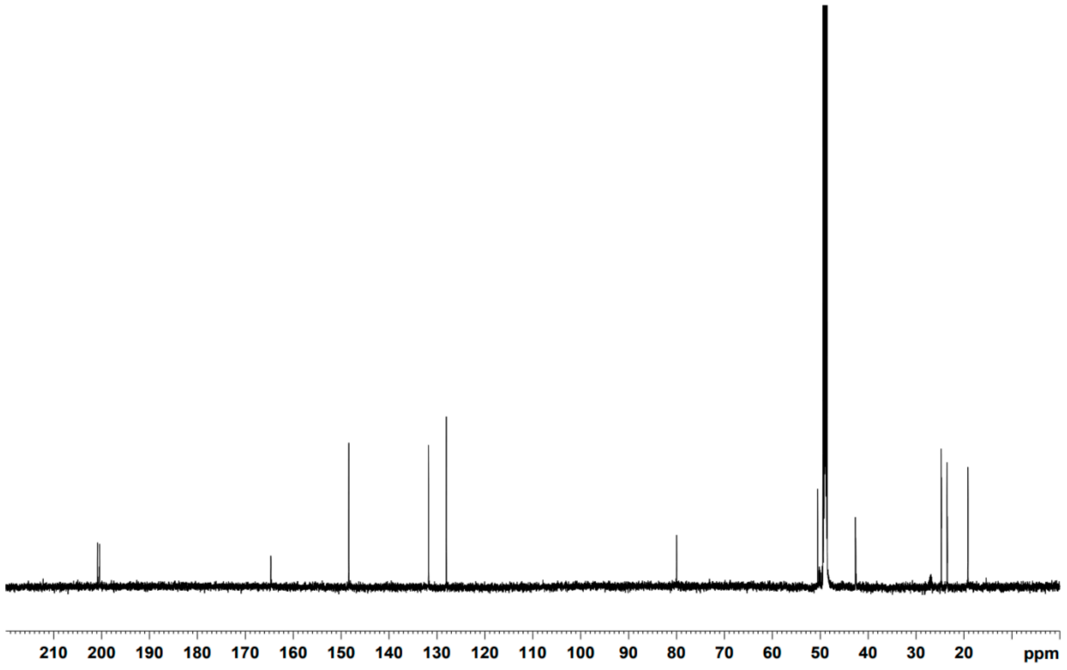

Figure S22.

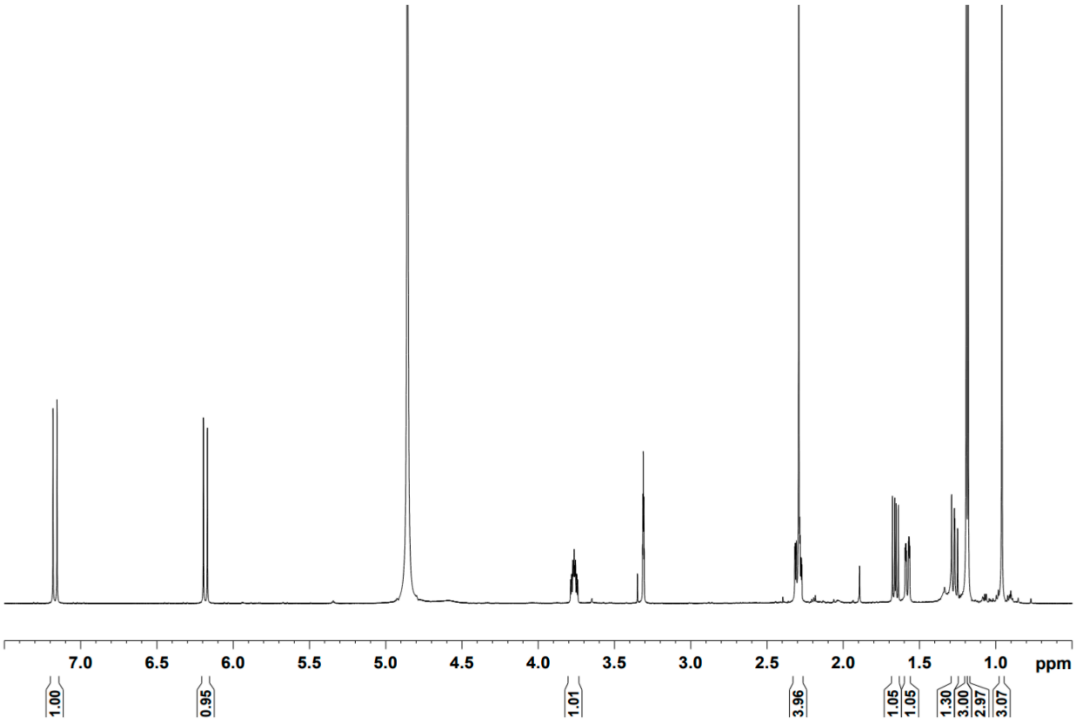

Figure S23.

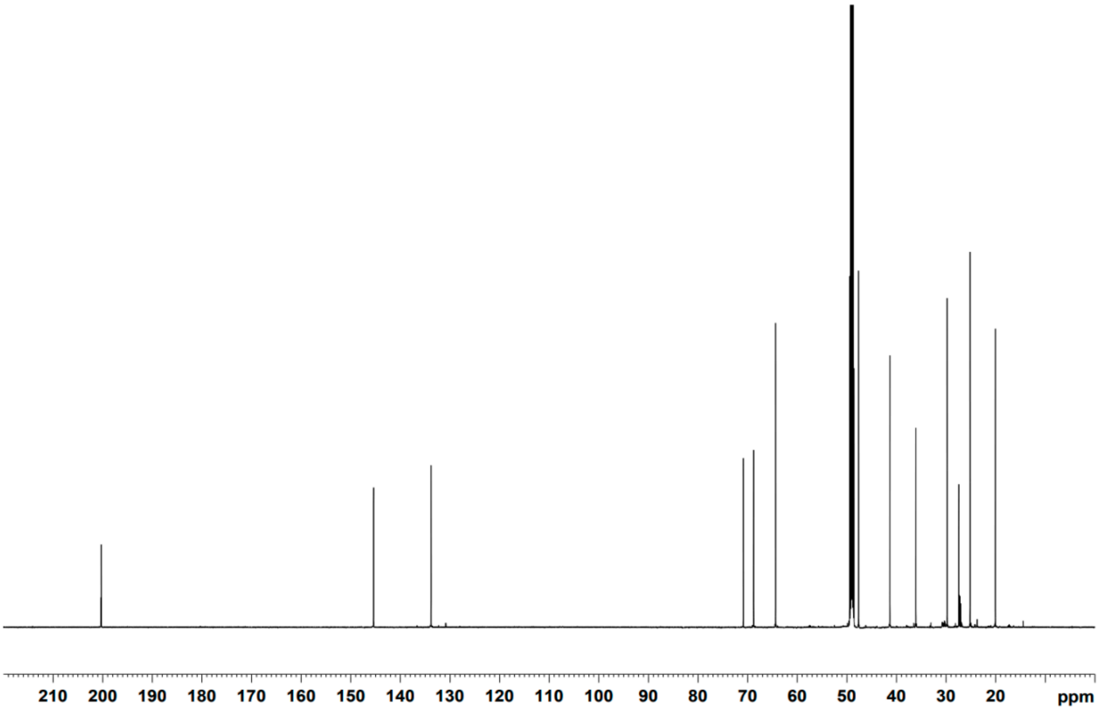

Figure S24.

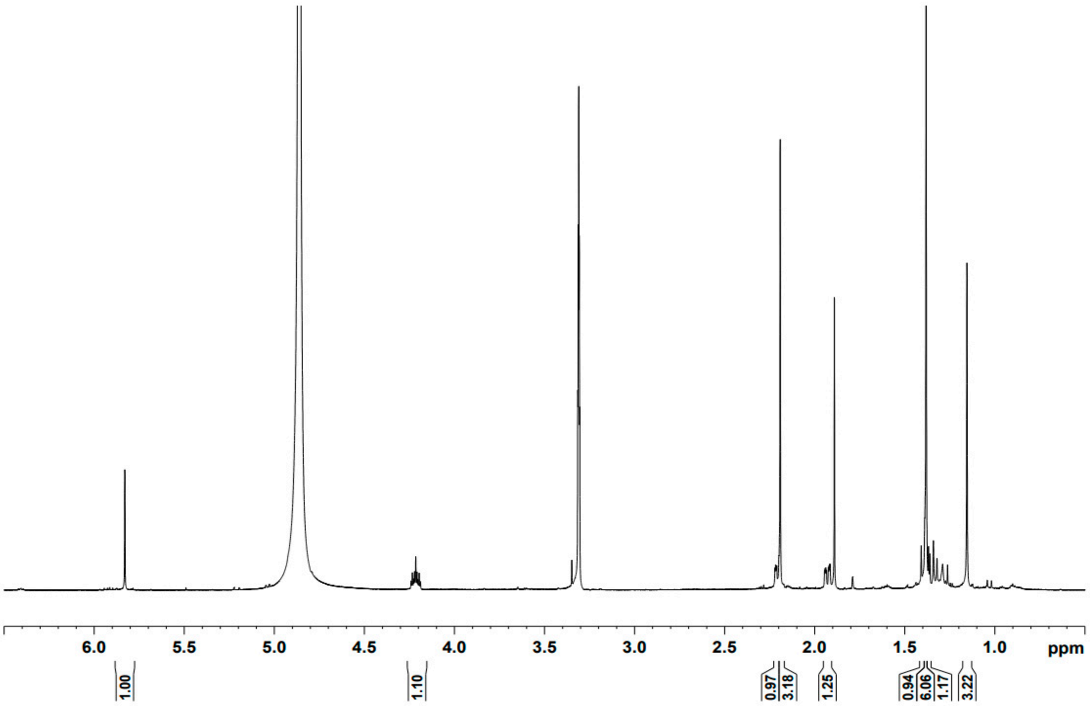

Figure S25.

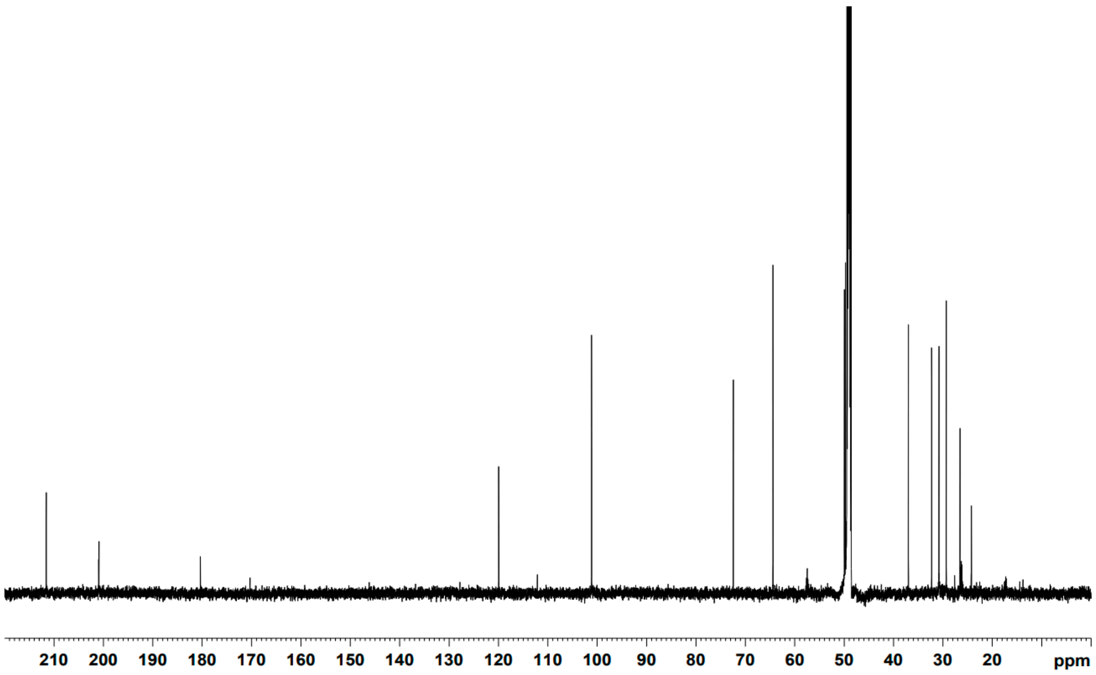

Figure S26.

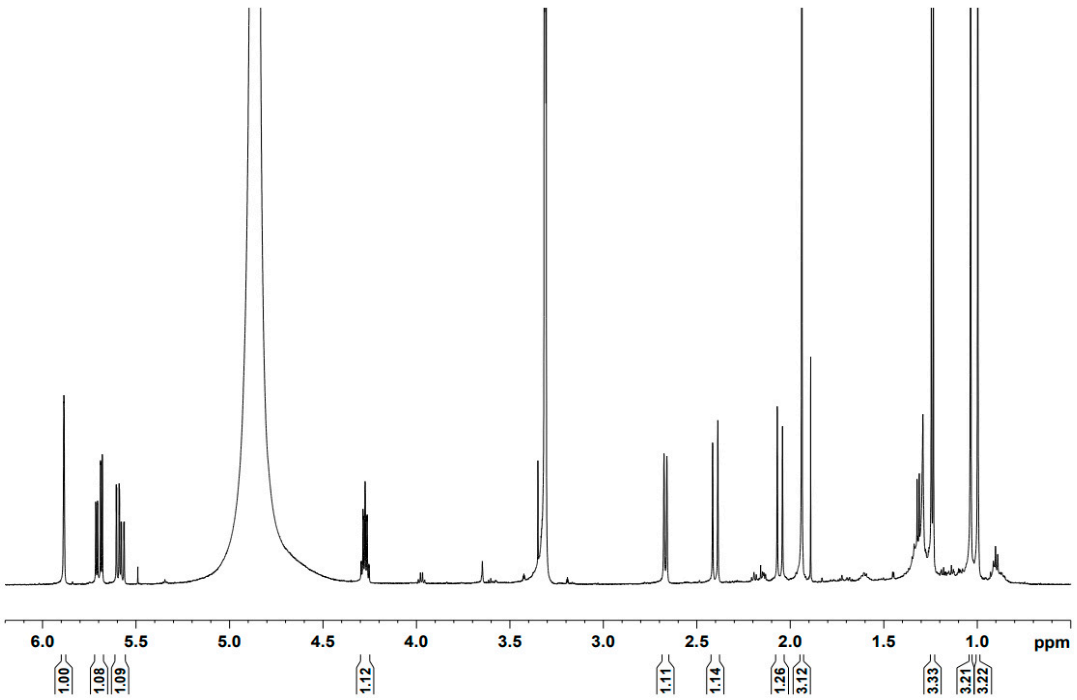

Figure S27.

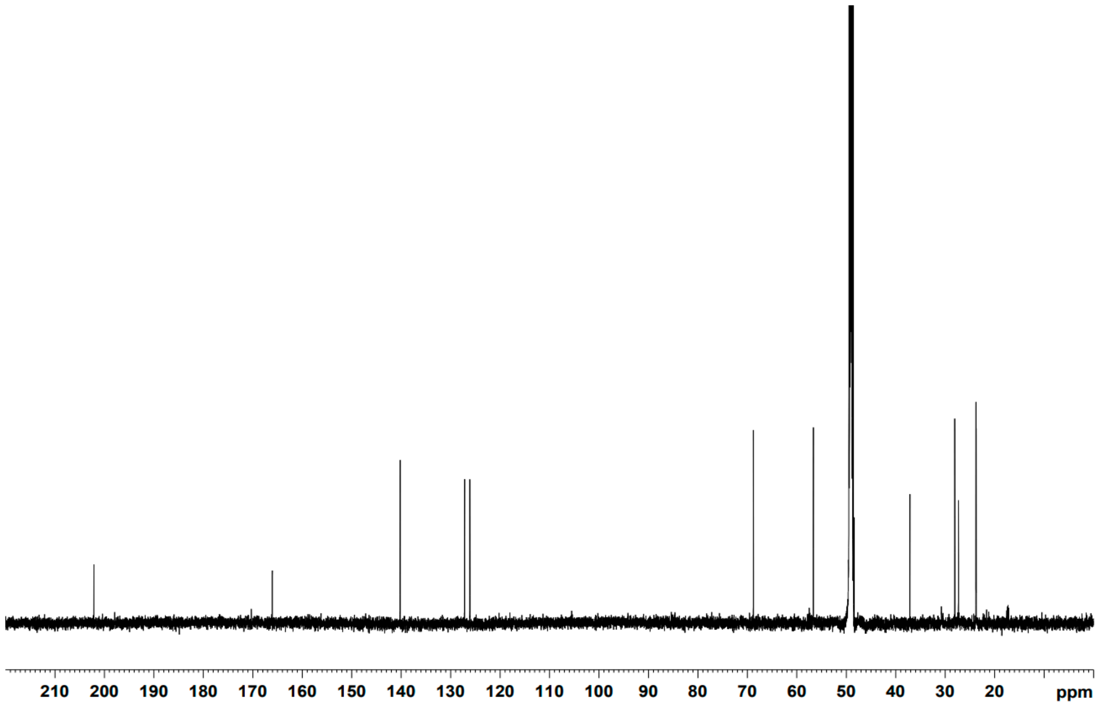

Figure S28.

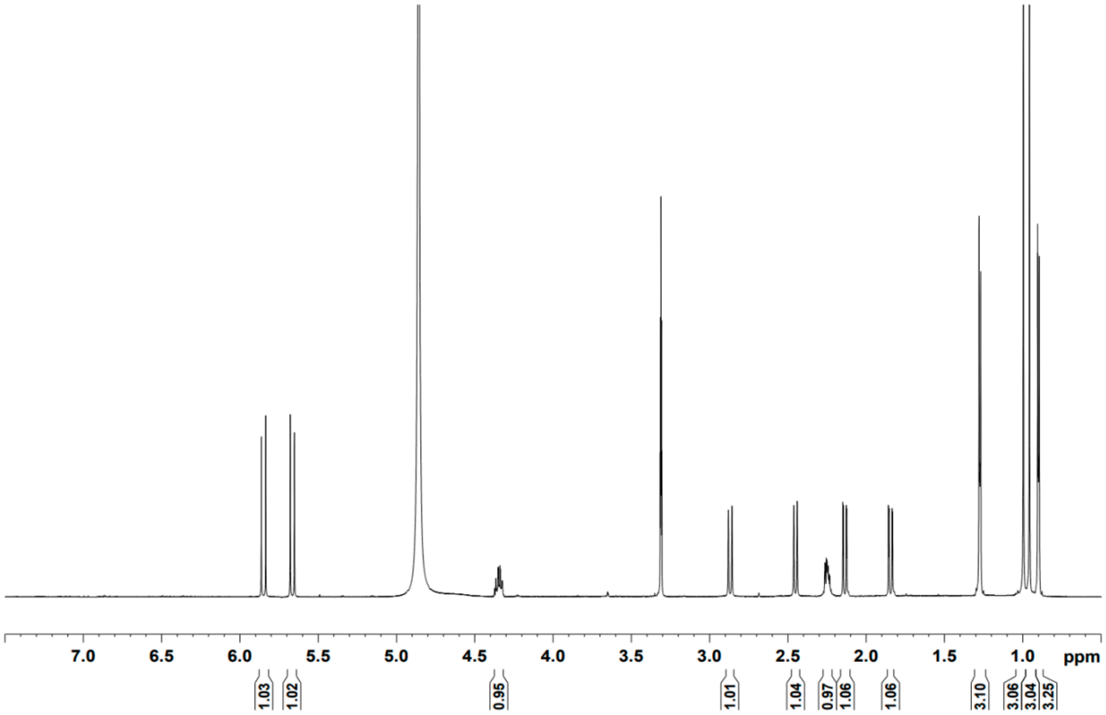

Figure S29.

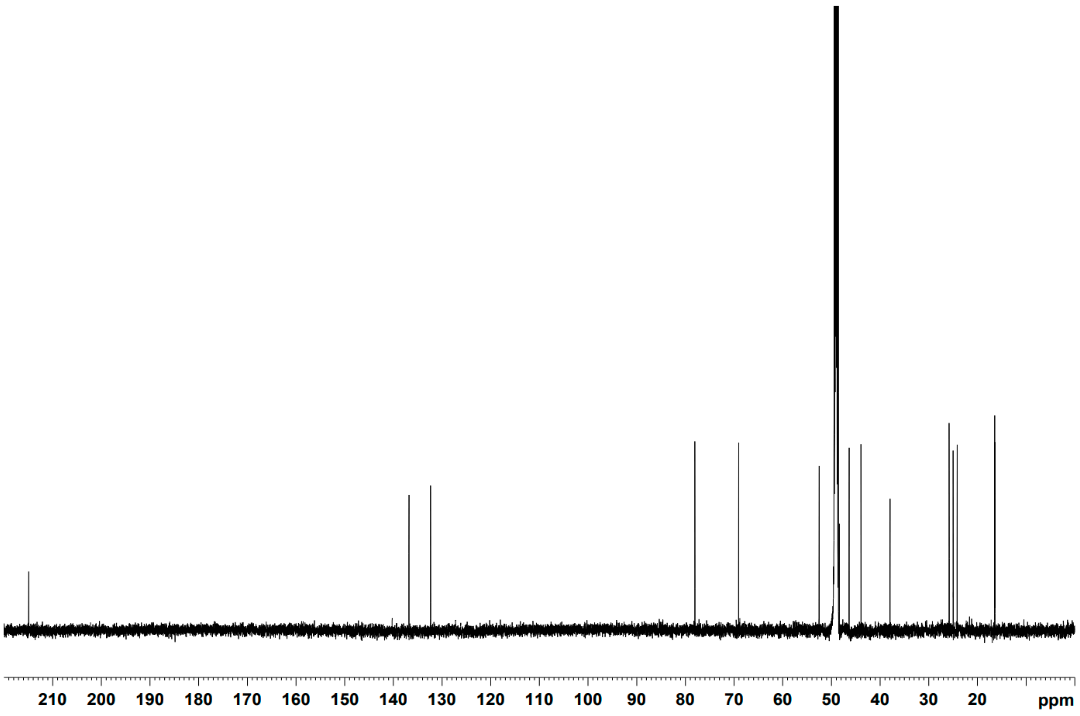

Figure S30.

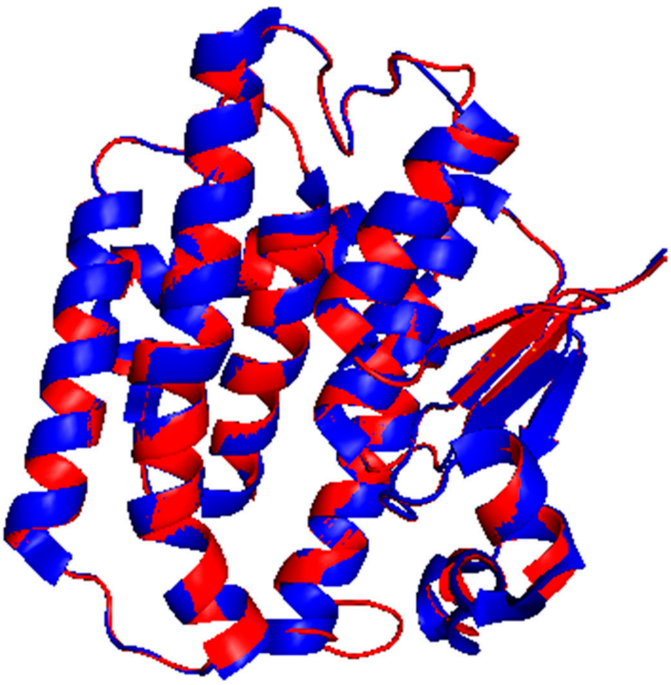

Figure S31.

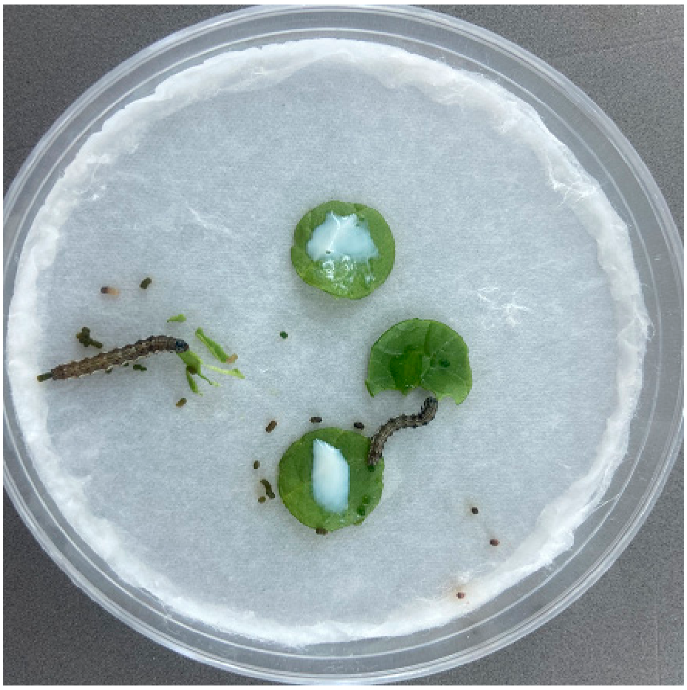

Figure S32.

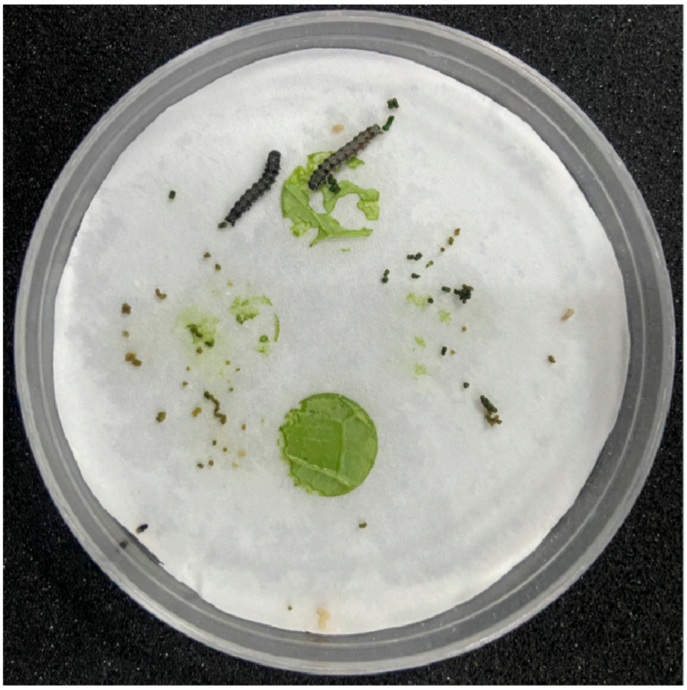

Figure S33.

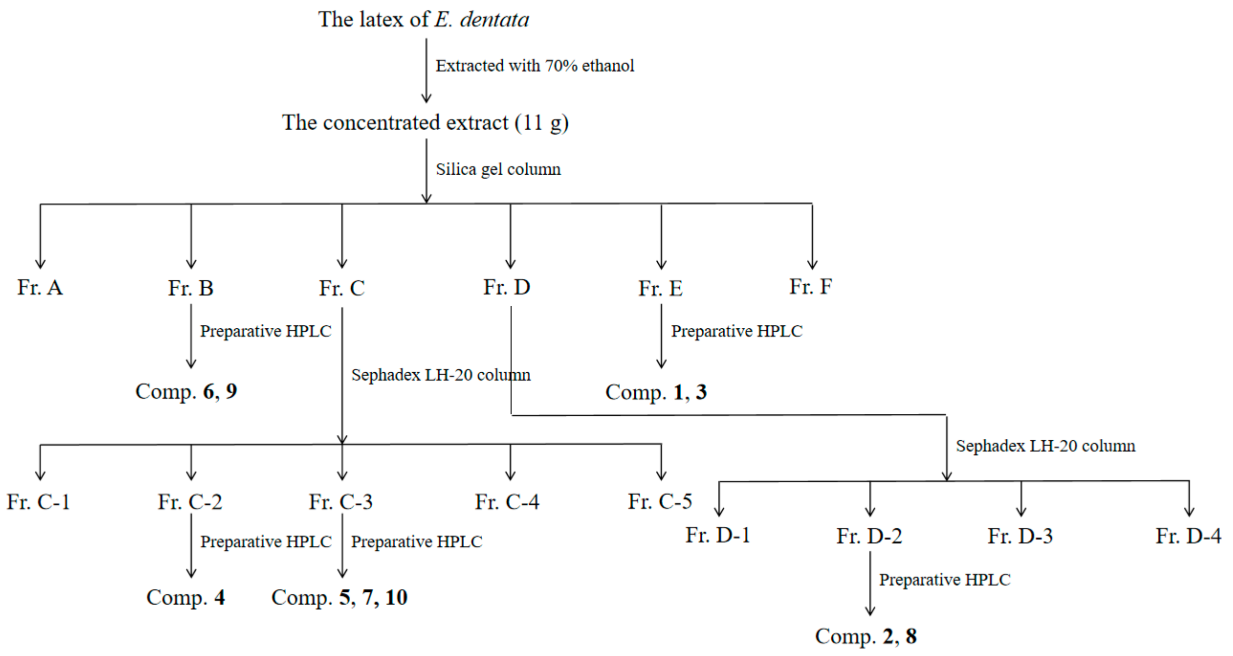

Table S1.

Template	Description	Sequence	PDB ID	E-Value	Sequence identity	Query coverage
GST	Chain A, Crystal Structure of Delta-Class Glutathione Transferase from Silkmoth	MSLDLYYAPGSAPCRVVLLV AAALDVHFNPHILNLRNGEH LTPEFLKLNPQHTVPTLVDG DFSLWESRAIGKYLVNKYGG ENNDLYPSDPKARAIVDQRL DFDLGTLYPRFGNYIYPQIFG GAKADEALLKKLEEALHFLN TFLEGQKYAAGDKLTLADLS LVATVSTIDAVDISLKEYPNV EKWFELVKATAPGYQEANE AGLKAFRAMVAQLKAKTEL	3VK9	8e-94	62.21%	98.0%

Table S2.

Ligand	Protein (GST)	
	Moldock Score (kcal/mol)	RMSD
**1**	−75.8056	4.7608
**2**	−82.0594	5.2135
**3**	−60.6084	4.1732
